# Enhanced nnU-Net Architectures for Automated MRI Segmentation of Head and Neck Tumors in Adaptive Radiation Therapy

**DOI:** 10.1007/978-3-031-83274-1_3

**Published:** 2025-03-03

**Authors:** Jessica Kächele, Maximilian Zenk, Maximilian Rokuss, Constantin Ulrich, Tassilo Wald, Klaus H. Maier-Hein

**Affiliations:** 1 German Cancer Research Center (DKFZ), Heidelberg, Germany; 2 Medical Faculty Heidelberg, University of Heidelberg, Heidelberg, Germany; 3 German Cancer Consortium (DKTK), DKFZ, core center Heidelberg, Heidelberg, Germany; 4 National Center for Tumor Diseases (NCT), NCT Heidelberg, A partnership between DKFZ and University Medical Center Heidelberg, Heidelberg, Germany; 5 Helmholtz Imaging, DKFZ, Heidelberg, Germany; 6 Faculty of Mathematics and Computer Science, Heidelberg University, Heidelberg, Germany; 7 Pattern Analysis and Learning Group, Department of Radiation Oncology, Heidelberg University Hospital, Heidelberg, Germany

**Keywords:** Head and Neck Cancer, MRI-guided Radiation Therapy, Tumor Segmentation, nnU-Net, Transfer Learning, Longitudinal Data Integration

## Abstract

The increasing utilization of MRI in radiation therapy planning for head and neck cancer (HNC) highlights the need for precise tumor segmentation to enhance treatment efficacy and reduce side effects. This work presents segmentation models developed for the HNTS-MRG 2024 challenge by the team mic-dkfz, focusing on automated segmentation of HNC tumors from MRI images at two radiotherapy (RT) stages: before (pre-RT) and 2–4 weeks into RT (mid-RT). For Task 1 (pre-RT segmentation), we built upon the nnU-Net framework, enhancing it with the larger Residual Encoder architecture. We incorporated extensive data augmentation and applied transfer learning by pre-training the model on a diverse set of public 3D medical imaging datasets. For Task 2 (mid-RT segmentation), we adopted a longitudinal approach by integrating registered pre-RT images and their segmentations as additional inputs into the nnU-Net framework. On the test set, our models achieved mean aggregated Dice Similarity Coefficient (aggDSC) scores of 81.2 for Task 1 and 72.7 for Task 2. Especially the primary tumor (GTVp) segmentation is challenging and presents potential for further optimization. These results demonstrate the effectiveness of combining advanced architectures, transfer learning, and longitudinal data integration for automated tumor segmentation in MRI-guided adaptive radiation therapy.

## 1 Introduction

Radiation therapy (RT) is fundamental in cancer treatment, with head and neck cancer (HNC) being one of the cancers that benefit most from this treatment. Recently, MRI-guided RT planning has gained traction due to MRI’s superior soft tissue contrast compared to traditional CT-based approaches [1].

Despite the potential of MRI-guided adaptive RT, the extensive volume of MRI data presents significant challenges. Manual segmentation of tumors by physicians, the current clinical standard, is often impractical due to time constraints and the complex nature of HNC tumors [2,3]. This has spurred interest in artificial intelligence (AI) approaches to automate and improve the accuracy of tumor segmentation [4]. Recent advancements in AI-driven segmentation have been propelled by public data challenges like the HECKTOR [5] and SegRap [6] Challenges, which have fostered collaborative innovations in the field. However, there remains a lack of large, publicly available AI-ready datasets specifically tailored for adaptive RT in HNC, limiting the broader adoption and clinical translation of these technologies.

To address this gap, the annual Medical Image Computing and Computer Assisted Intervention Society (MICCAI) Head and Neck Tumor Segmentation for MR-Guided Applications (HNTS-MRG) 2024 challenge has been established, focusing on the segmentation of HNC tumors for MRI-guided adaptive RT applications. A unique aspect of HNTS-MRG 2024 is its emphasis on evaluating whether incorporating prior timepoint data into auto-segmentation algorithms enhances performance for RT applications.

This work presents the results of our segmentation models applied to the HNTS-MRG 2024 MRI training and testing datasets. For pre-radiotherapy (pre-RT) images, we utilized ResEnc models within the nnU-Net framework [7], enhanced through transfer learning and ensembling. Additionally, we developed a model for mid-radiotherapy (mid-RT) images that leverages the pre-RT images and their segmentations.

## 2 Methods

### 2.1 Imaging Data

The dataset employed in this study, made available for the HNTS-MRG 2024 Challenge, includes pre-RT and mid-RT MRI scans from 200 patients with HNC. This collection is divided into 150 cases for training and 50 cases for testing, as designated by the challenge organizers. Each training case is accompanied by consensus ground truth segmentations of the GTVp and GTVn regions, which were generated by multiple clinical experts. Additionally, the pre-RT MRI images and their corresponding segmentations are provided in both their original form and after being registered to the mid-RT images.

For the segmentation of pre-RT images, only the pre-RT MRI scans were utilized during the training process. In contrast, the segmentation of mid-RT images incorporated both pre-RT and mid-RT MRI scans along with the pre-RT segmentations, thereby enhancing model performance.

### 2.2 Task 1

Our approach for Task 1 builds upon the well-established nnU-Net framework [8], leveraging the enhanced capabilities of the larger ResEnc architecture preset as described in [7].

#### nnU-Net Configuration.

For our experiments, we employed the 3d_fullres configuration of the nnU-Net framework. The input images were resampled to the median spacing of [1.12, 0.5, 0.5] mm, and intensities were normalized using Z-score normalization. The network was trained over 1000 epochs with a batch size of 2, utilizing an input patch size of 64×320×256. The default nnU-Net settings were applied for the learning rate, optimizer, and loss function.

#### Training Variants.

Upon the baseline default nnU-Net and ResEnc models, we trained the following variants:

1. **extensive augmentations (Aug++) model:** We further enhanced the data augmentation strategy by incorporating additional and stronger augmentations beyond those provided by nnU-Net. These augmentations were designed to expose the model to a wider range of data variations, thereby improving its robustness and generalization capabilities. Notably, local intensity transformations such as patch blacking, sharpening, Gaussian intensity gradients, and local gamma adjustments significantly expanded the diversity of patches. Due to the spatial constraints in this manuscript, we refer to [9] for the full details and augmentation scheme. Moreover, an implementation of said scheme is included in the nnU-Net v2 repository as nnUNetTrainerDA5 [8]. The model was implemented using the ResEnc M U-Net architecture [7].
2. **pretrained model:** To incorporate prior knowledge and enhance our model’s capabilities, we pre-trained it on an extensive and diverse collection of 3D medical images, combining various public datasets in a manner inspired by MultiTalent [10]. During this pretraining phase, we utilized separate segmentation heads for each dataset to accommodate their specific characteristics. The model was trained for 4000 epochs with a patch size of 192×192×192 and a batch size of 24 distributed to 8xA100 GPUs. A batch size of 24 was selected as it represents the maximum capacity supported by the available GPUs. Due to the dataset’s substantial size, 4000 epochs were necessary to ensure the model’s convergence, in line with findings from [11]. All images were resampled to an isotropic resolution of 1×1×1 mm^3^ per voxel and normalized using Z-score normalization.

To ensure balanced training across datasets of different sizes, we sampled datasets inversely proportional to the square root of the number of images in each dataset. This approach prevented larger datasets from dominating the training process. The model was implemented using the nnU-Net framework [8] and the ResEnc L U-Net architecture [7].

The pretrained model weights are made publicly available^1^. A comprehensive overview of the datasets used for pretraining can be found in Table 4. The respective model was not explicitly trained and optimized for this challenge. After completing the pretraining phase, we fine-tuned the model on the HNTSMRG 2024 dataset. The initial preprocessing configuration was maintained, featuring a patch size of 64×320×256 and a spacing of [1.12, 0.5, 0.5] mm. We introduced an additional 50 epochs to warm up the network. During this warm-up period, the learning rate was linearly increased until it reached a learning rate of 1 × 10^−3^, allowing for a smoother transition and better convergence during fine-tuning. After the warm-up, the default polynomial learning rate schedule with an initial learning rate of 1 × 10^−3^ was used.

#### Ensembling.

We employed an ensembling strategy of multiple models to enhance the performance and robustness of our segmentation model. By averaging the predicted probabilities for each voxel across the best-performing models, we combined their strengths. This probabilistic averaging mitigates individual model biases and uncertainties, leading to more accurate and reliable segmentation results.

#### Postprocessing.

Postprocessing steps were applied to refine the segmentation results. We utilized connected component analysis to remove low-volume predictions. Specifically, any connected components corresponding to GTVn with a volume less than 675 mm^3^ and GTVp with a volume less than 750 mm^3^ were removed from the final segmentation.

#### Test-Time Augmentation.

Due to increased inference time associated with ensembling models, we considered reducing test-time augmentation (TTA). To assess whether this reduction would lead to worse performance compared to using a single model with TTA, we turned off TTA when evaluating the best-performing ensemble on the validation folds. This allowed us to determine if the ensembling without TTA could still perform competitively within the time constraints.

#### Alternative Approaches Explored.

We experimented with several alternative approaches that did not yield improved results. Training the model on both pre-RT and mid-RT images resulted in a decline in performance, possibly because the mid-RT images introduced information that confused or misled the model during training.

We also attempted to align the preprocessing steps with those used during pretraining to ensure consistency between the datasets. This involved using a patch size of 192×192×192, and resampling the images to a spacing of [1, 1, 1] mm. While these modifications led to a higher DSC, the aggDSC worsened. This suggests that the model’s performance improved on images with low tumor volumes, which do not have a substantial influence on the aggDSC.

Additionally, we experimented with batch dice loss, calculating the aggDSC for each batch to closely mimic the evaluation metric. However, this approach did not enhance performance.

#### Final Test Set Submission.

Based on the results from 5-fold cross-validation, we chose to ensemble the Aug++ and the pretrained model for testing on the test set. During inference, we combined the models obtained from each of the five training folds and both models by averaging their softmax probabilities for each voxel. To comply with the 20-minute time constraint per case, we reduced TTA to mirroring along only one axis instead of all three axes typically used by nnU-Net.

### 2.3 Task 2

#### Method Overview.

During the development phase, we evaluated a range of segmentation models. The baseline model is nnU-Net, which operates on a single input image. For training, we explored two approaches: using either only mid-RT scans or sampling minibatches from the combined set of mid-RT and pre-RT scans, while using the corresponding mask (mid-RT or pre-RT) as the target. In the latter case, the training and validation sets were split to ensure that scans from the same patient were not divided across sets. We refer to these models as **nnU-Net (only mid)** and **nnU-Net (pre & mid)** in the task-2 results.

Our main approach also builds on the nnU-Net framework but integrates longitudinal information by using both registered pre- and mid-RT scans as input, following the methodology of LongiSeg [12]. In short, this method predicts the segmentation mask of a *current* scan by using additional imaging data from a *prior* scan, which is acquired at an earlier time and provides context for improving the prediction of the current time point. We implemented the following “LongiSeg” variants:

1. **Base model:** Pre-RT and mid-RT scans are concatenated along the channel dimension and input into the network. The assignment of which scan serves as the current scan and which as the prior is randomized and the concatenation order is (current, prior). The network then outputs the segmentation of the current scan. This approach enhances the diversity of the training data but does not preserve the chronological order. The loss is computed with the segmentation of the current scan.
2. **SkipDiff:** This variant also encodes prior and current images separately using encoders with shared weights. Feature maps at each U-Net stage are merged using a difference weighting block and fed via skip connections into a single decoder, which generates the segmentation of the current image. Difference weighting multiply the current feature maps with the normalized difference between current and prior features, to capture the changes between the pre- and mid-RT scans, which brought performance gains in [12].
3. **Pre-seg:** In addition to the image inputs, a one-hot encoding of the registered prior scan segmentation mask is added to the network input, resulting in the order (current, prior, prior mask). Two variations of this method were tested: In the first variant (**pre-seg**), current and prior scans were assigned randomly to pre-RT and mid-RT, as for the base model. In the second variant (**pre-seg-c**), training followed the chronological order, so that the mid-RT scan is always used as current scan and pre-RT as prior scan.

#### nnU-Net Configuration.

All models were implemented in the nnU-Net framework and used the 3d_fullres configuration. The input images were resampled to a spacing of [1.12, 0.5, 0.5] mm, and image intensities were normalized using Z-score normalization. The network was trained with a batch size of 2 for 1000 epochs (250k minibatch updates), using an input patch size of 48×224×192. The loss function was a sum of batch DSC and cross-entropy loss. The default nnU-Net settings were applied for the data augmentations, learning rate, and optimizer. The U-Net architecture was adapted dynamically to the dataset by nnU-Net and had six stages. We also tried the residual encoder U-Net in preliminary experiments, but it did not improve results.

#### Final Test Set Submission.

Based on 5-fold cross-validation, the model LongiSeg: **pre-seg-c** was selected as the submission for the final test set evaluation. During inference, we ensembled the models obtained from each of the 5 training folds by averaging their softmax probabilities. Additionally, for each model, test-time augmentation with 3 mirroring augmentations was applied, so that 4 predictions are averaged for each model. We reduced the number of mirroring operations from the default of 7 to 3, to stay within the inference time limits.

## 3 Results

The results presented originate from the evaluation on the validation set of the 5-fold cross-validation. For Task 2, only the mid-RT scans were taken into account, as they are the targets during testing as well. As metrics, the mean DSC and aggDSC were used for choosing the final model and are reported. For both the average per class was calculated and then aggregated across the classes. 90% confidence intervals for aggDSC were estimated with the bootstrapping method, using 1000 bootstrap samples.

### 3.1 Task 1

As shown in Table 1, the ResEnc architectures outperformed the baseline nnU-Net, with the ResEnc M architecture slightly surpassing the ResEnc L architecture. Through more data augmentation, the DSC improved marginally (from 0.621 to 0.624), although the aggregated aggDSC decreased slightly (from 0.821 to 0.82).

**Fig. 1.**
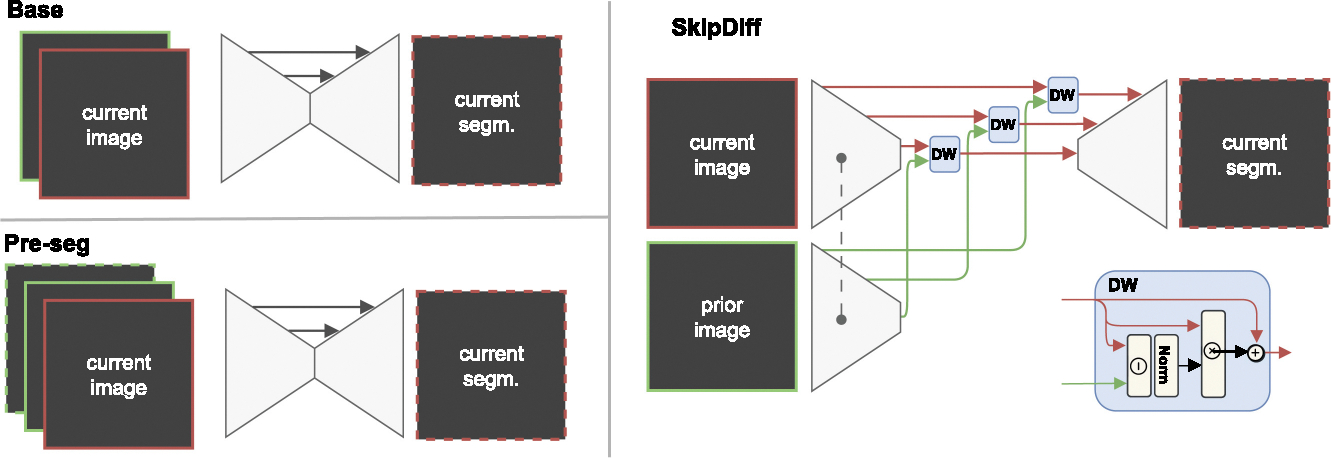
Overview of the three longitudinal segmentation models developed for task 2. Red boxes indicate the data for the current time point, and green boxes for a prior time. Images have solid outlines and segmentations have dashed lines (the third spatial dimension is not shown for clarity). The *Base* model stacks the current image and prior images in the channel dimension, and the *Pre-seg* model also stacks the prior segmentation. *SkipDiff* uses a shared encoder for prior and current images; the features are merged with a difference weighting block (DW). Adapted from [12].

Pretraining led to a boost in performance, pushing the aggDSC from 0.814 to 0.823. Postprocessing steps contributed to a modest enhancement in performance, increasing the aggDSC to 0.824. The best results, with an aggDSC of 0.828, were achieved by ensembling the Aug++ model in conjunction with the pretrained model. Figure 1 presents representative samples from the validation folds, illustrating the overlay of tumor mask outlines on MRI images. It was observed that ensembling without TTA still performed better than a single model with TTA. This finding justifies the use of an ensembled model despite the computational constraints.

The results of the additional experiments discussed in Sect. 2.2 are presented in Table 5 in the appendix.

#### Test Set Results.

On the official test set, our model achieved a mean aggDSC of 0.812, with individual aggDSC values of 0.869 for GTVn and 0.756 for GTVp. Compared to our cross-validation results, the GTVn score on the test set shows a slight improvement, whereas the GTVp score is considerably lower.

### 3.2 Task 2

#### Initial Model Exploration.

We initially focused on fast experimentation, testing a variety of models on a single training fold. The results from these preliminary experiments are presented in Table 2. The LongiSeg pre-seg model, which leveraged both the registered pre-RT scan and its corresponding mask, outperformed all others in this phase. While the LongiSeg base model achieved slightly better performance than the best nnU-Net model, the difference is likely not statistically significant. Notably, there was a substantial performance gap between the nnU-Net trained solely on mid-RT scans and the one trained on both pre-RT and mid-RT scans, highlighting the advantage of a larger training dataset. Based on these findings, we decided to concentrate on the LongiSeg models, particularly the pre-seg variant and its potential optimizations.

#### Cross-Validation Results.

**Fig. 2.**
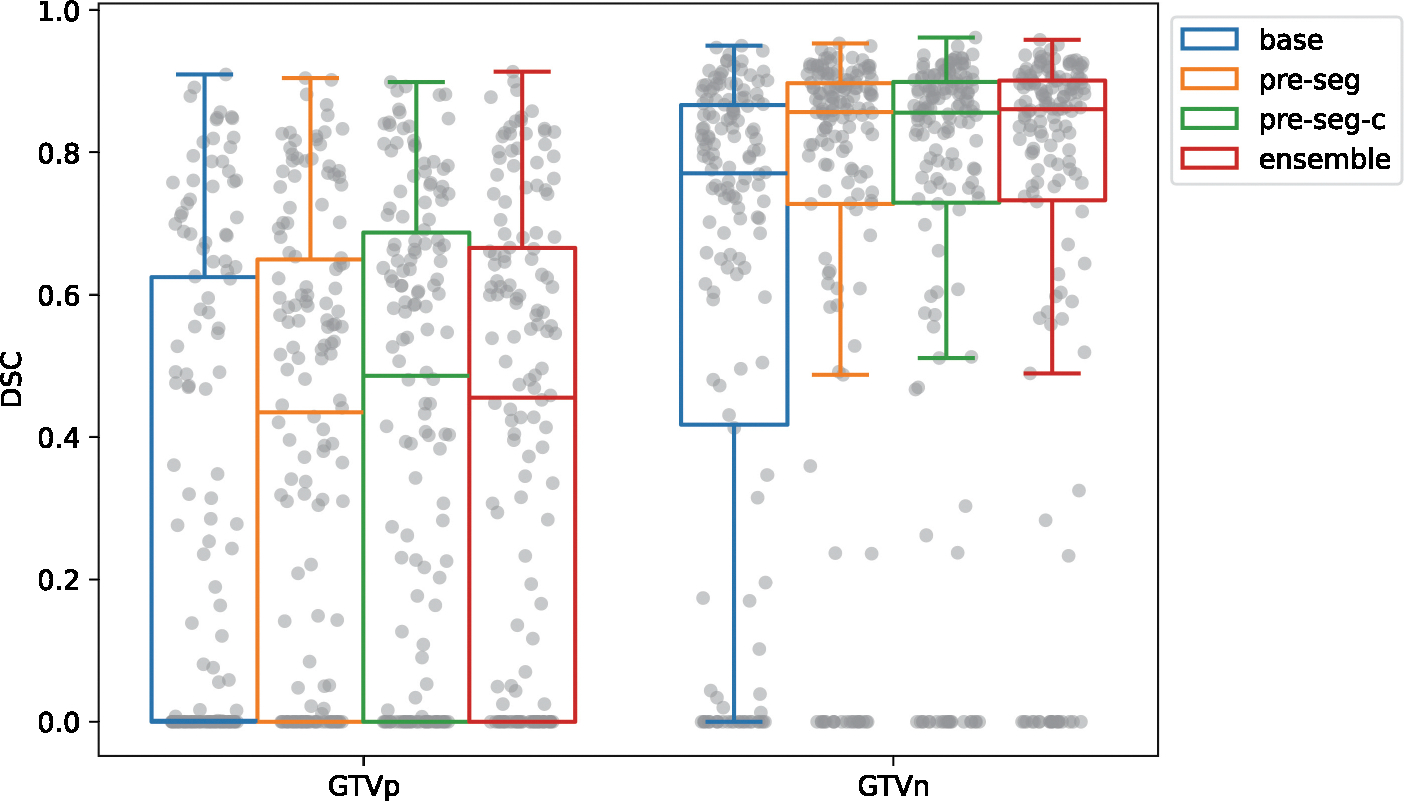
Individual DSC scores for each model in the cross-validation evaluation for task 2. A box plot summarizes the quartiles of each distribution. Cases with zero DSC occur especially if the ground truth is empty and the prediction is not, or vice versa.

The results from the 5-fold cross-validation are shown in Table 3. In the appendix, Fig. 2 reports DSC values for individual cases. We also trained the pre-seg-c variant, which maintained the chronological order of the scans during training. This variant improved the aggDSC for the GTVp region compared to the pre-seg model, although the performance on the GTVn region saw a slight decline. Both models significantly outperformed the LongiSeg base model. While we considered an ensemble of the two best models for our final submission, its aggDSC scores were lower in cross-validation. Therefore, we selected the LongiSeg pre-seg-c model as our final choice.

#### Test Set Results.

On the official test set for task 2, our model achieved a mean aggDSC score of 0.7268, with individual aggDSC values of 0.8747 for GTVn and 0.5789 for GTVp. Compared to our cross-validation results, the GTVn score is as expected, while the GTVp score is considerably lower.

## 4 Conclusion

In this work, we developed and evaluated segmentation models for the segmentation of HNC tumors in MRI-guided adaptive radiation therapy applications, as part of the HNTS-MRG 2024 challenge. Our approaches, grounded in the nnU-Net framework and enhanced through techniques such as transfer learning, ensembling, and longitudinal data integration, demonstrated significant improvements over baseline models.

Our models achieved mean aggDSC scores of 0.812 for Task 1 and 0.727 for Task 2 on the test set, performing slightly worse than on the validation folds. While the performance for the GTVn region remained robust, the GTVp segmentation exhibited a notable drop compared to cross-validation results. Possibly there are more difficult cases in the test set or outliers that affect the test set scores. In general, the GTVp is significantly harder to segment, as shown by the higher inter-rater variability [13], which may also contribute to statistical noise in test set results. A closer analysis of failure cases on the validation and test sets could inspire application-specific refinements to enhance GTVp segmentation.

For future work, leveraging separate segmentations from individual raters would be beneficial to account for inter-rater variability, which is particularly high for GTVp. Furthermore, employing a probabilistic U-Net [14] could help manage segmentation uncertainties, enabling the model to capture a broader range of anatomical variations that appear to be present in GTVp.

Moreover, our exploration into reducing inference time indicates that optimizing preprocessing pipelines-for instance, by implementing data-specific preprocessing-can facilitate the deployment of larger models and enable more extensive ensembling without compromising computational efficiency.

## Figures and Tables

**Table 1.** Task 1 results for 5-fold cross-validation (150 validation cases) for different nnU-Net models trained on pre-RT scans, computed using the official evaluation code. aggDSC is used in the challenge ranking and a confidence interval is estimated with 1000 bootstrap samples. DSC is given as an auxiliary metric, computed on each case individually including cases with empty ground truth mask, too.

Setting	aggDSC			DSC
	GTVp	GTVn	mean	mean

**Baseline**				
nnU-Net	78.0 [75.6,80.8]	84.9 [83.0,87.0]	81.4 [79.7,83.3]	61.5

**Model Architecture: ResEnc M**				
nnU-Net ResEnc M	78.6 [76.2,81.4]	85.6 [83.9,87.5]	82.1 [80.5,84.0]	62.1
+ extensive augmentations (Aug++)	78.1 [75.8,80.9]	85.9 [84.4,87.7]	82.0 [80.5,83.8]	62.4

**Model Architecture: ResEnc L**				
nnU-Net ResEnc L	77.3 [74.9,80.2]	85.4 [83.6,87.5]	81.4 [79.7,83.3]	61.4
+ Pretraining	78.7 [76.4,81.5]	85.9 [84.3,87.8]	82.3 [80.8,84.0]	62.6
+ Postprocessing	78.7 [76.3,81.3]	86.0 [84.3,87.8]	82.4 [80.8,84.0]	62.3

**Ensembling**				
Aug++ model + pretrained model	**79.0** [76.8,81.4]	**86.5** [85.0,88.4]	**82.8** [81.3,84.3]	**62.7**

**Without TTA**				
Aug++ model + pretrained model	78.8 [76.6,81.8]	86.1 [85.5,88.0]	82.5 [80.9,84.3]	**62.7**

**Table 2.** Metric results on a single training fold (fold 0, 30 validation cases) for a range of compared segmentation models, computed using the official evaluation code. aggDSC is used in the challenge ranking and a confidence interval is estimated with 1000 boot-strap samples. DSC is given as an auxiliary metric, computed on each case individually including cases with empty ground truth mask, too.

Model	aggDSC			DSC
	GTVp	GTVn	mean	mean

nnU-Net: only mid	51.5 [36.0, 67.8]	81.1 [75.7, 84.0]	66.3 [58.4, 75.5]	42.49
nnU-Net pre & mid	57.0 [43.0, 71.0]	82.8 [77.8, 85.8]	69.9 [62.2, 76.5]	47.10
LongiSeg: base	58.1 [45.1, 70.9]	82.8 [77.6, 86.2]	70.4 [63.4, 77.1]	47.46
LongiSeg: skipdiff	53.3 [44.8, 61.3]	83.4 [78.8, 86.9]	68.4 [63.5, 73.3]	48.56
LongiSeg: pre-seg	**61.5** [53.6, 71.6]	**88.0** [85.8, 89.5]	**74.8** [70.6, 79.9]	**56.25**

**Table 3.** Metric results for 5-fold cross-validation (150 validation cases) for different LongiSeg models, computed using the official evaluation code. aggDSC is used in the challenge ranking and a confidence interval is estimated with 1000 bootstrap samples. DSC is given as an auxiliary metric, computed on each case individually including cases with empty ground truth mask, too.

Model	aggDSC			DSC
	GTVp	GTVn	mean	mean

LongiSeg: base	54.9 [49.0, 60.6]	82.5 [80.3, 84.7]	68.7 [65.7, 71.9]	43.87
LongiSeg: pre-seg	61.1 [56.8, 65.8]	87.5 [86.2, 88.8]	74.3 [72.3, 76.9]	54.65
LongiSeg: pre-seg-c	**64.1** [59.8, 68.2]	87.3 [85.9, 88.5]	**75.7** [73.5, 78.1]	**55.92**
Ensemble pre-seg; -c	63.3 [59.1, 67.9]	**87.7** [86.4, 89.0]	75.5 [73.5, 78.0]	—

## Footnotes

1 https://zenodo.org/records/13753413.

## A Appendix

**Table 4.** Overview over all datasets used for the supervised pretraining

Name	Images	Modality	Target	Link

Decathlon Task 3 [15, 16]	131	CT	Liver, L. Tumor	http://medicaldecathlon.com/
Decathlon Task 6 [15, 16]	63	CT	Lung Lesion	http://medicaldecathlon.com/
Decathlon Task 7 [15, 16]	281	CT	Pancreas, P. Tumor	http://medicaldecathlon.com/
Decathlon Task 8 [15, 16]	303	CT	Hepatic Vessel, H. Tumor	http://medicaldecathlon.com/
Decathlon Task 9 [15, 16]	41	CT	Spleen	http://medicaldecathlon.com/
Decathlon Task 10 [15, 16]	126	CT	Colon Tumor	http://medicaldecathlon.com/
BTCV [17]	30	CT	13 abdominal organs	https://www.synapse.org/Synapse:syn3193805/wiki/89480
LIDC [18]	1010	CT	Lung lesion	https://www.cancerimagingarchive.net/collection/lidc-idri/
BTCV 2 [19]	63	CT	9 abdominal organs	https://zenodo.org/records/1169361#.YiDLFnXMJFE
StructSeg Task1 [20]	50	CT	22 OAR Head & neck	https://structseg2019.grand-challenge.org
StructSeg Task2 [20]	50	CT	Nasopharynx cancer	https://structseg2019.grand-challenge.org/Home/
StructSeg Task3 [20]	50	CT	6 OAR Lung	https://structseg2019.grand-challenge.org/Home/
StructSeg Task4 [20]	50	CT	Lung Cancer	https://structseg2019.grand-challenge.org/Home/
SegTHOR [21]	40	CT	Heart, aorta, trachea, esophagus	https://competitions.codalab.org/competitions/21145
NIH-Pan [22–24]	82	CT	Pancreas	https://wiki.cancerimagingarchive.net/display/Public/Pancreas-CT
VerSe2020 [25–27]	113	CT	28 Vertebrae	https://github.com/anjany/verse
RibSeg [28]	370	CT	Rips	https://github.com/M3DV/RibSeg?tab=readme-ov-file
KiTs23 [29]	489	CT	Kidneys, k. Tumors, Cysts	https://kits-challenge.org/kits23/
AbdomenAtlas1.0 [30, 31]	5195	CT	8 abdominal organs	https://github.com/MrGiovanni/AbdomenAtlas?tab=readme-ov-file
TotalSegmentatorV2 [11]	1180	CT	117 classes of whole body	https://github.com/wasserth/TotalSegmentator
FLARE [32]	50	CT	13 abdominal organs	https://flare22.grand-challenge.org/
SegRap [33]	120	CT	45 OARs (Head&Neck)	https://segrap2023.grand-challenge.org/
SegA [34–36]	56	CT	Aorta	https://multicenteraorta.grand-challenge.org/data/
WORD [37, 38]	120	CT	16 abdominal organs	https://github.com/HiLab-git/WORD
AbdomenCT1K [39]	996	CT	Liver, Kidney, Spleen, pancreas	https://github.com/JunMa11/AbdomenCT-1K
DAP-ATLAS [40]	533	CT	142 classes of whole body	https://github.com/alexanderjaus/AtlasDataset
CTORG [41]	140	CT	Lung, brain, bones, liver, kidneys and bladder	https://www.cancerimagingarchive.net/collection/ct-org/
HanSeg [42]	42	CT	OAR (Head&Neck)	https://han-seg2023.grand-challenge.org/
Decathlon Task 2 [15, 16]	20	MRI	Heart	http://medicaldecathlon.com/
Decathlon Task 4 [15, 16]	208	MRI	Hippocampus	http://medicaldecathlon.com/
Decathlon Task 5 [15, 16]	32	MRI	Prostate	http://medicaldecathlon.com/
ISLES2015 [43]	28	MRI	Stroke Lesion	http://www.isles-challenge.org/ISLES2015/
Promise12 [44]	50	MRI	Prostate	https://zenodo.org/records/8026660
ACDC [45]	200	MRI	RV cavity, myocardium, LV cavity	https://www.creatis.insa-lyon.fr/Challenge/acdc/databases.html
ISBILesion2015 [46]	42	MRI	MS Lesion	https://iacl.ece.jhu.edu/index.php/MSChallenge
CHAOS [47]	60	MRI	Liver, Kidney (L&R), Spleen	https://zenodo.org/records/3431873
M&Ms [48, 49]	300	MRI	L. ventricle, R. ventricle, L. ventri. myocardium	https://www.ub.edu/mnms/
ProstateX [50]	140	MRI	Prostate lesion	https://www.aapm.org/GrandChallenge/PROSTATEx-2/
MSLesion [51]	48	MRI	MS Lesion	https://data.mendeley.com/datasets/8bctsm8jz7/1
BrainMetShare [52]	84	MRI	Brain Metastases	https://aimi.stanford.edu/brainmetshare
CrossModa22 [53]	168	MRI	Vestibular schwannoma, cochlea	https://crossmoda2022.grand-challenge.org/
Atlas22 [54]	524	MRI	Stroke lesion	https://atlas.grand-challenge.org/
BraTs23 [55–58]	1251	MRI	Glioblastoma	https://www.synapse.org/Synapse:syn51156910/wiki/621282
AutoPet2 [59]	1014	PET, CT	Lesions	https://autopet-ii.grand-challenge.org/
Hecktor2022 [60]	524	PET, CT	Nodal Gross Tumor Volumes (Head&Neck)	https://hecktor.grand-challenge.org/
AMOS [61]	360	CT, MRI	15 abdominal organs	https://amos22.grand-challenge.org/
TopCow [62]	200	CT, MRI	Vessel components of CoW	https://topcow23.grand-challenge.org/

**Table 5.** Task 1 -fold cross-validation results for different nnU-Net configurations, that did not yield improved results. Settings include training on pre & mid-RT images, applying pretraining preprocessing, and incorporating batch dice.

Setting	aggDSC			DSC
	GTVp	GTVn	mean	mean
pre & mid-RT	75.6 [73.0,78.5]	84.3 [82.4,86.4]	79.9 [78.9,81.8]	61.0
pretraining preprocessing	78.6 [76.5,81.1]	85.6 [83.8,87.9]	82.1 [80.5,83.9]	63.6
batch dice	78.7 [76.3,81.5]	85.5 [83.7,87.6]	82.1 [80.4,84.0]	61.1

## References

[1] Pollard JM, Wen Z, Sadagopan R, Wang J, Ibbott GS: The future of image-guided radiotherapy will be MR guided. Br. J. Radiol. 90(1073), 20160667 (2017) 28256898 10.1259/bjr.20160667 PMC5605101

[2] Thorwarth D, Low DA: Technical challenges of real-time adaptive MR-guided radiotherapy. Front. Oncol. 11, 634507 (2021) 33763369 10.3389/fonc.2021.634507 PMC7982516

[3] Segedin B, Petric P: Uncertainties in target volume delineation in radiotherapy - are they relevant and what can we do about them? Radiol. Oncol. 50(3), 254–262 (2016) 27679540 10.1515/raon-2016-0023 PMC5024655

[4] Hindocha S, : Artificial intelligence for radiotherapy auto-contouring: current use, perceptions of and barriers to implementation. Clin. Oncol. (R Coll. Radiol.) 35(4), 219–226 (2023) 36725406 10.1016/j.clon.2023.01.014

[5] Oreiller V, : Head and neck tumor segmentation in PET/CT: the HECKTOR challenge. Med. Image Anal. 77(102336), 102336 (2022) 35016077 10.1016/j.media.2021.102336

[6] Luo X, : Segrap2023: a benchmark of organs-at-risk and gross tumor volume segmentation for radiotherapy planning of nasopharyngeal carcinoma (2023) 10.1016/j.media.2024.103447 39756265

[7] Isensee F, : nnu-net revisited: a call for rigorous validation in 3D medical image segmentation (2024)

[8] Isensee F, Jaeger PF, Kohl SA, Petersen J, Maier-Hein KH: nnU-Net: a self-configuring method for deep learning-based biomedical image segmentation. Nat. Methods 18(2), 203–211 (2021) 33288961 10.1038/s41592-020-01008-z

[9] Pflüger I, : Automated detection and quantification of brain metastases on clinical MRI data using artificial neural networks. Neurooncol. Adv. 4(1), vdac138 (2022) 36105388 10.1093/noajnl/vdac138 PMC9466273

[10] Ulrich C, Isensee F, Wald T, Zenk M, Baumgartner M, Maier-Hein KH: Multitalent: a multi-dataset approach to medical image segmentation. In: Medical Image Computing and Computer Assisted Intervention - MICCAI 2023: 26th International Conference, Vancouver, BC, Canada, 8–12 October 2023, Proceedings, Part III, pp. 648–658. Springer, Heidelberg (2023)

[11] Wasserthal J, : Totalsegmentator: robust segmentation of 104 anatomic structures in CT images. Radiol. Artif. Intell. (2023) 10.1148/ryai.230024 PMC10546353 37795137

[12] Rokuss M, : Longitudinal segmentation of MS lesions via temporal difference weighting (2024)

[13] Lin D, : E pluribus unum: prospective acceptability benchmarking from the contouring collaborative for consensus in radiation oncology crowdsourced initiative for multiobserver segmentation. J. Med. Imaging 10(S1), S11903–S11903 (2023) 10.1117/1.JMI.10.S1.S11903 PMC9907021 36761036

[14] Kohl SAA, : A probabilistic u-net for segmentation of ambiguous images. In: NIPS 2018, pp. 6965–6975. Curran Associates Inc, Red Hook (2018)

[15] Antonelli M, : The medical segmentation decathlon. arXiv:2106.05735 (2021)

[16] Simpson AL, : A large annotated medical image dataset for the development and evaluation of segmentation algorithms. arXiv:1902.09063 (2019)

[17] Landman B, Zhoubing X, Igelsias JE, Styner M, : MICCAI multi-atlas labeling beyond the cranial vault workshop and challenge. In: Proceedings of MIC-CAI Multi-Atlas Labeling Beyond Cranial Vault-Workshop Challenge (2015)

[18] Armato SG III, : Data from LIDC-IDRI (2015)

[19] Gibson E, : Automatic multi-organ segmentation on abdominal CT with dense V-networks. IEEE Trans. Med. Imaging (2018) 10.1109/TMI.2018.2806309 PMC6076994 29994628

[20] Li H, Zhou J, Deng J, Chen M: Automatic structure segmentation for radiotherapy planning challenge (2019). https://structseg2019.grand-challenge.org/. Accessed 25 Feb 2022

[21] Lambert Z, Petitjean C, Dubray B, Kuan S: Segthor: segmentation of thoracic organs at risk in CT images. arXiv:1912.05950 (2019)

[22] Roth HR, : Deeporgan: multi-level deep convolutional networks for automated pancreas segmentation. arXiv:1506.06448 (2015)

[23] Clark K, : The cancer imaging archive (TCIA): maintaining and operating a public information repository. J. Digit. Imaging (2013) 10.1007/s10278-013-9622-7 PMC3824915 23884657

[24] Roth HR, : Deeporgan: multi-level deep convolutional networks for automated pancreas segmentation. In: Medical Image Computing and Computer-Assisted Intervention – MICCAI 2015 (2015)

[25] Sekuboyina A, : Verse: a vertebrae labelling and segmentation benchmark for multi-detector CT images. Med. Image Anal. (2021) 10.1016/j.media.2021.102166 34340104

[26] Löffler MT, : A vertebral segmentation dataset with fracture grading. Radiol. Artif. Intell. (2020) 10.1148/ryai.2020190138 PMC8082364 33937831

[27] Liebl H, : A computed tomography vertebral segmentation dataset with anatomical variations and multi-vendor scanner data (2021) 10.1038/s41597-021-01060-0 PMC8553749 34711848

[28] Yang J, Gu S, Wei D, Pfister H, Ni B: Ribseg dataset and strong point cloud baselines for rib segmentation from CT scans. In: de Bruijne M, (eds.) Medical Image Computing and Computer Assisted Intervention – MICCAI 2021 (2021)

[29] Heller N, : The KITS21 challenge: automatic segmentation of kidneys, renal tumors, and renal cysts in corticomedullary-phase CT (2023)

[30] Li W, Yuille A, Zhou Z: How well do supervised models transfer to 3D image segmentation? In: The Twelfth International Conference on Learning Representations (2024)

[31] Qu C, Zhang T, Qiao H, Tang Y, Yuille AL, Zhou Z: Abdomenatlas-8k: annotating 8,000 CT volumes for multi-organ segmentation in three weeks. In: Advances in Neural Information Processing Systems (2023)

[32] Ma J, : Unleashing the strengths of unlabeled data in pan-cancer abdominal organ quantification: the flare22 challenge. arXiv preprint arXiv:2308.05862 (2023) 10.1016/S2589-7500(24)00154-7 39455194

[33] Luo X, : SegRap2023: a benchmark of organs-at-risk and gross tumor volume segmentation for radiotherapy planning of NasoPharyngeal carcinoma. arXiv (2023) 10.1016/j.media.2024.103447 39756265

[34] Radl L, : AVT: multicenter aortic vessel tree CTA dataset collection with ground truth segmentation masks. Data Brief (2022) 10.1016/j.dib.2022.107801 PMC8760499 35059483

[35] Jin Y, : AI-based aortic vessel tree segmentation for cardiovascular diseases treatment: status quo. arXiv (2021)

[36] Pepe A, : Detection, segmentation, simulation and visualization of aortic dissections: a review. Med. Image Anal. (2020) 10.1016/j.media.2020.101773 32738647

[37] Luo X, : Word: a large scale dataset, benchmark and clinical applicable study for abdominal organ segmentation from CT image. Med. Image Anal. (2022) 10.1016/j.media.2022.102642 36223682

[38] Liao W, : Comprehensive evaluation of a deep learning model for automatic organs-at-risk segmentation on heterogeneous computed tomography images for abdominal radiation therapy. Int. J. Radiat. Oncol. Biol. Phys. 117(4), 994–1006 (2023) 37244625 10.1016/j.ijrobp.2023.05.034

[39] Ma J, : Abdomenct-1k: is abdominal organ segmentation a solved problem? IEEE Trans. Pattern Anal. Mach. Intell. (2022) 10.1109/TPAMI.2021.3100536 34314356

[40] Jaus A, : Towards unifying anatomy segmentation: automated generation of a full-body CT dataset via knowledge aggregation and anatomical guidelines. arXiv preprint arXiv:2307.13375 (2023)

[41] Rister B, Shivakumar K, Nobashi T, Rubin DL: CT-ORG: a dataset of CT volumes with multiple organ segmentations (2019) 10.1038/s41597-020-00715-8 PMC7658204 33177518

[42] Podobnik G, Strojan P, Peterlin P, Ibragimov B, Vrtovec T: HaN-Seg: the head and neck organ-at-risk CT and MR segmentation dataset. Med. Phys. (2023) 10.1002/mp.16197 36594372

[43] Maier O, : Extra tree forests for sub-acute ischemic stroke lesion segmentation in MR sequences. J. Neurosci. Methods (2015) 10.1016/j.jneumeth.2014.11.011 25448384

[44] Litjens G, : Evaluation of prostate segmentation algorithms for MRI: the promise12 challenge. Med. Image Anal. (2014) 10.1016/j.media.2013.12.002 PMC4137968 24418598

[45] Bernard O, : Deep learning techniques for automatic MRI cardiac multi-structures segmentation and diagnosis: is the problem solved? IEEE Trans. Med. Imaging (2018) 10.1109/TMI.2018.2837502 29994302

[46] Carass A, : Longitudinal multiple sclerosis lesion segmentation: resource and challenge. NeuroImage (2017) 10.1016/j.neuroimage.2016.12.064 PMC5344762 28087490

[47] Kavur AE, Selver MA, Dicle O, Barış M, Gezer NS: CHAOS -combined (CT-MR) healthy abdominal organ segmentation challenge data (2019) 10.1016/j.media.2020.101950 33421920

[48] Campello VM, : Multi-centre, multi-vendor and multi-disease cardiac segmentation: the MMS challenge. IEEE Trans. Med. Imaging (2021) 10.1109/TMI.2021.3090082 34138702

[49] Martín-Isla C, : Deep learning segmentation of the right ventricle in cardiac MRI: the MMS challenge. IEEE J. Biomed. Health Inform. (2023) 10.1109/JBHI.2023.3267857 37067963

[50] Litjens G, Debats O, Barentsz J, Karssemeijer N, Huisman H: Spie-aapm prostatex challenge data (2017)

[51] Muslim AM: Brain MRI dataset of multiple sclerosis with consensus manual lesion segmentation and patient meta information (2022) 10.1016/j.dib.2022.108139 PMC9043670 35496484

[52] Grøvik E, Yi D, M. Iv, Tong E, Rubin D, Zaharchuk G: Deep learning enables automatic detection and segmentation of brain metastases on multisequence MRI. J. Magn. Reson. Imaging (2020) 10.1002/jmri.26766 PMC7199496 31050074

[53] Shapey J, : Segmentation of vestibular schwannoma from magnetic resonance imaging: an open annotated dataset and baseline algorithm (vestibular-schwannoma-SEG) (2021) 10.1038/s41597-021-01064-w PMC8553833 34711849

[54] Liew S-L, : A large, open source dataset of stroke anatomical brain images and manual lesion segmentations. Sci. Data (2018) 10.1038/sdata.2018.11 PMC5819480 29461514

[55] Karargyris A, : Federated benchmarking of medical artificial intelligence with medperf. Nat. Mach. Intell. (2023) 10.1038/s42256-023-00652-2 PMC11068064 38706981

[56] Bakas S, : Advancing the cancer genome atlas glioma MRI collections with expert segmentation labels and radiomic features. Sci. Data (2017) 10.1038/sdata.2017.117 PMC5685212 28872634

[57] Menze BH, : The multimodal brain tumor image segmentation benchmark (BRATS). IEEE Trans. Med. Imaging (2015) 10.1109/TMI.2014.2377694 PMC4833122 25494501

[58] Baid U, : The RSNA-ASNR-MICCAI BraTS 2021 benchmark on brain tumor segmentation and radiogenomic classification. arXiv (2021)

[59] Gatidis S, Kuestner T: A whole-body FDG-PET/CT dataset with manually annotated tumor lesions (FDG-PET-CT-Lesions) (2022) 10.1038/s41597-022-01718-3 PMC9532417 36195599

[60] Andrearczyk V, : Overview of the HECKTOR challenge at MICCAI 2022: automatic head and neck TumOR segmentation and outcome prediction in PET/CT. Head Neck Tumor Chall. (2022) (2023) 10.1007/978-3-031-27420-6_1 PMC10171217 37195050

[61] Ji Y, : Amos: a large-scale abdominal multi-organ benchmark for versatile medical image segmentation. In: Advances in Neural Information Processing Systems (2022)

[62] Yang K, : Benchmarking the cow with the topcow challenge: topology-aware anatomical segmentation of the circle of willis for CTA and MRA (2024)

